# A microfluidic culture model of the human reproductive tract and 28-day menstrual cycle

**DOI:** 10.1038/ncomms14584

**Published:** 2017-03-28

**Authors:** Shuo Xiao, Jonathan R. Coppeta, Hunter B. Rogers, Brett C. Isenberg, Jie Zhu, Susan A. Olalekan, Kelly E. McKinnon, Danijela Dokic, Alexandra S. Rashedi, Daniel J. Haisenleder, Saurabh S. Malpani, Chanel A. Arnold-Murray, Kuanwei Chen, Mingyang Jiang, Lu Bai, Catherine T. Nguyen, Jiyang Zhang, Monica M. Laronda, Thomas J. Hope, Kruti P. Maniar, Mary Ellen Pavone, Michael J. Avram, Elizabeth C. Sefton, Spiro Getsios, Joanna E. Burdette, J. Julie Kim, Jeffrey T. Borenstein, Teresa K. Woodruff

**Affiliations:** 1 Department of Obstetrics and Gynecology, Feinberg School of Medicine, Northwestern University, Chicago, Illinois 60611, USA; 2 The Charles Stark Draper Laboratory, Cambridge, Massachusetts 02139, USA; 3 Ligand Assay and Analysis Core, Center for Research in Reproduction, University of Virginia, Charlottesville, Virginia 22908, USA; 4 Department of Pathology, Feinberg School of Medicine, Northwestern University, Chicago, Illinois 60611, USA; 5 Department of Anesthesiology, Feinberg School of Medicine, Northwestern University, Chicago, Illinois 60611, USA; 6 Department of Dermatology, Feinberg School of Medicine, Northwestern University, Chicago, Illinois 60611, USA; 7 Department of Medicinal Chemistry and Pharmacognosy, University of Illinois at Chicago, Chicago, Illinois 60607, USA

## Abstract

The endocrine system dynamically controls tissue differentiation and homeostasis, but has not been studied using dynamic tissue culture paradigms. Here we show that a microfluidic system supports murine ovarian follicles to produce the human 28-day menstrual cycle hormone profile, which controls human female reproductive tract and peripheral tissue dynamics in single, dual and multiple unit microfluidic platforms (Solo-MFP, Duet-MFP and Quintet-MPF, respectively). These systems simulate the *in vivo* female reproductive tract and the endocrine loops between organ modules for the ovary, fallopian tube, uterus, cervix and liver, with a sustained circulating flow between all tissues. The reproductive tract tissues and peripheral organs integrated into a microfluidic platform, termed EVATAR, represents a powerful new *in vitro* tool that allows organ–organ integration of hormonal signalling as a phenocopy of menstrual cycle and pregnancy-like endocrine loops and has great potential to be used in drug discovery and toxicology studies.

## Abstract (web summary)

The female reproductive tract constitutes the ovary, fallopian tubes, uterus, and cervix, but it is challenging to engineer this system *in vitro*. Here, the authors develop a microfluidic device (EVATAR) with reproductive tract and peripheral tissues to replicate hormone release of a 28-day menstrual cycle.

The female reproductive tract is required for the production of ova, secretion of sex hormones and the maintenance of pregnancy throughout the gestation of healthy offspring. Entry and advancement through puberty, normal menstrual cycles with a potential intervening pregnancy, and endocrine support of peripheral tissues, such as the bone, brain and heart, are all essential roles that the reproductive tract tissues and their hormones play. The main organs of the female reproductive tract are the ovaries, fallopian tubes, uterus and cervix. Each organ has major responsibilities that are either autonomous (for example, maturation of oocytes and gestation of the fetus) or interdependent such as providing hormonal support for the tracts through which gametes (eggs and sperm) travel and a location for the developing embryo to implant. Moreover, each organ is composed of cells from multiple lineages (for example, myometrium and endometrium in the uterus) that provide local function and feedback control. Because of the intimate relationship between the cells of each organ and between organs, toxicology studies in the female reproductive tract have been difficult to design.

Methods used to grow mammalian cells outside the body have not fundamentally changed in the last 50 years. Preclinical studies often begin with individual cells, separated from cellular and physical contacts that are important for biological function^1^. These dispersed cells must be propagated through weekly reduction divisions and maintained on flat plastic; however, these cells are missing the cell physicochemical microenvironment, three-dimensional (3D) tissue-specific architecture, and blood flow perfusion found in natural tissues. Furthermore, typical media composition is based on basal nutrients, bovine serum and a few specialized factors that are placed in a static setting with random mixing. As a consequence, cell–cell and tissue-level cytokine and endocrine signals are not integrated into signalling pathways. In parallel with these developments, the pharmaceutical industry is challenged by the fact that fewer drugs are emerging to address many unmet needs, including cardiovascular disease, cancer, immune diseases, and new contraceptives^2^,^3^,^4^. Despite large investments in research funding, only ∼8% of drugs for which Investigational New Drug applications have been filed will be approved by the FDA^5^. Innovative methods to culture cells *in vitro* to test new compounds are therefore necessary to reinvigorate the drug pipeline.

Recently, organ-on-a-chip and human-on-a-chip microfluidic technologies have garnered significant interest and offer promising approaches to test the efficacy and toxicity of new drugs *in vitro*^16^,^17^,^18^. Microfluidics represents an engineered manipulation of fluid flow in a set of micrometre-sized channels and provides precise control of microlitre volume of fluids. In the current study, Solo-MFP and Duet-MFP systems based on pneumatic actuation technology and a Quintet-MFP system based on embedded electromagnetic actuation technology were designed for single and multiple tissue cultures, respectively. Mouse ovarian tissue was cultured in the Solo-MFP and Duet-MFP systems for 28 days, which resulted in follicle production of 28-day menstrual cycle hormone profiles. To test the ovarian hormone control of downstream human female reproductive tract and peripheral tissues, the ovary, fallopian tube, uterus, cervix and liver were cultured in the Quintet-MFP system. This integrated microfluidic platform enables dynamic and precisely controlled interaction between organs and is operational over the course of month-long experiments. This represents a next step in fundamental and applied toxicology as well as therapeutic discovery and deployment to address a wide range of biological problems and fill the drug pipeline.

## Results

### Microfluidic technology enabled dynamic tissue integration

**Figure 1:**
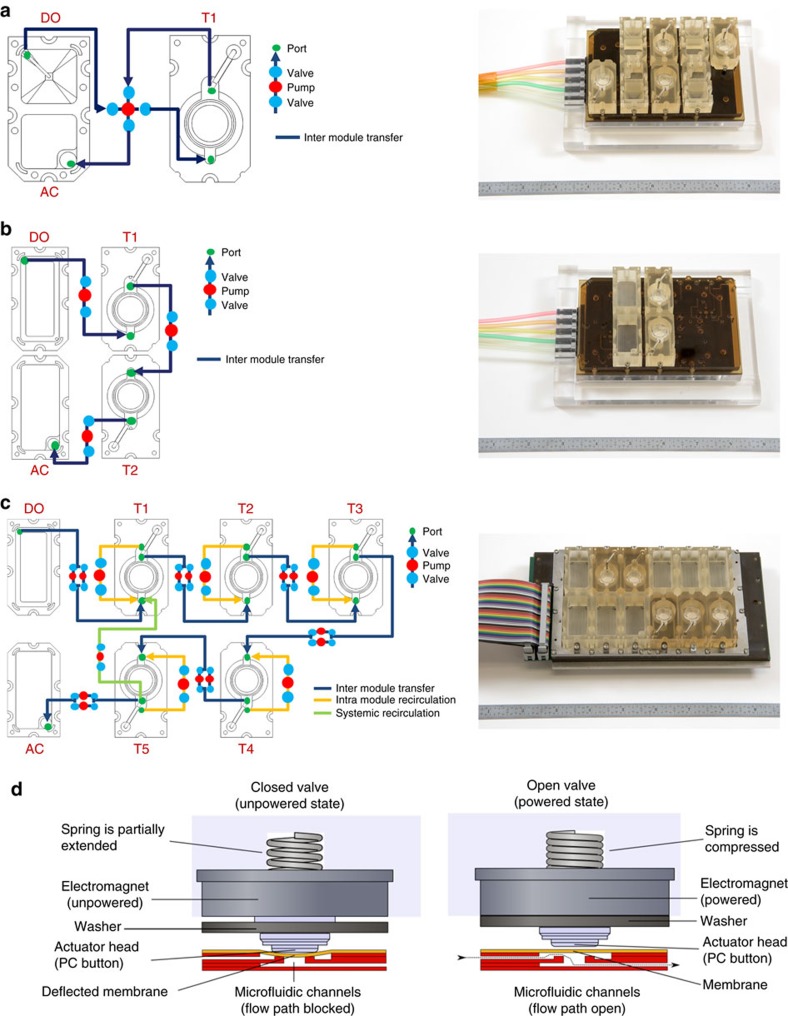
Microfluidic platform design. Digital images and flow diagrams illustrating the pump pathways of the (**a**) Solo-MFP, (**b**) Duet-MFP and (**c**) Quintet-MFP systems. (**d**) Illustration of the pump mechanism of the electromagnetic Quintet-MFP. AC, acceptor module; DO, donor module; T, tissue module.

The first step of our work was to develop platforms that could sustain tissue-level function for the length of the human menstrual cycle (that is, 28 days). The Solo-MFP and Duet-MFP systems are based on pneumatic actuation technology, by which the individual systems are supplied with positive and negative air pressures via a system dock that is connected to a five-channel pressure controller manifold (Fig. 1a,b, refer to Supplementary Table 1 for nomenclature). The pressure of individual channels is switched between a vacuum or pressure source using an electromagnetic three-way valve controlled via a personalized pump programme created in LabVIEW using a computer interface. The Solo-MFP and Duet-MFP systems use a universal pneumatic plate that distributes positive and negative air pressures to specific valve or pump membranes clamped between the pneumatic and fluidic plates. Sequential application of pressure and vacuum to valve and pump membranes creates a peristalsis-like stroke that enables fluid to move through individual microfluidic paths within or between modules. Four valves arranged in North, South, East and West positions that are oriented about a central pump chamber create a four-port pump structure, enabling multiple bi-directional flows in each fluidic circuit. The fluidic plates are specifically designed for either the Solo-MFP or Duet-MFP module configuration. Solo-MFP systems consist of four replicates of a fluidic circuit with two connected modules: a coupled donor/acceptor module and a module for tissues (Fig. 1a). Duet-MFP systems consist of a single set of four modules: a donor module, two modules for tissues, and a separate acceptor module (Fig. 1b). Pneumatic actuation manipulates the membranes, generating pressure-driven flow in the fluidic paths, in order to transport fresh media from the donor to tissues, remove older media and secreted factors to the acceptor, or (in the case of Duet-MFP systems) move fluid from the upstream tissue to the downstream tissue to enable *in vitro* communication between cultured tissues.

To integrate five tissues in a single system, a more practical and scalable approach was used for microfluidic control of tissue interaction (Fig. 1c). This was accomplished by embedding electromagnetically actuated micropumps within the platform, termed the Quintet-MFP, obviating the need to supply the platform with independent air lines for each pneumatic actuator (Fig. 1d). This design approach allowed each of the 60 actuators of the Quintet-MFP to be individually controlled, thus enabling precise flow control over a wide dynamic range. Modules included flow ports to permit recirculation within each module, ensuring that the system was well mixed and enabling homogenous exposure of cultured tissues to factors within the media. In addition to recirculation within individual modules, the fluidic path design allowed for whole-system recirculation (Fig. 1c). The combination of whole-system and intermodule recirculation enabled a well-mixed system within and across all modules of the Quintet-MFP. Each fluidic path between modules was controlled by two sets of pump actuators. This redundancy acted as a fail-safe in the event of actuator failure. A total of twelve modules, including one donor, one acceptor, five tissue-specific modules, and five blank modules (for increased tissue capacity in future work) were developed for the Quintet-MFP (Fig. 1c). As with the Solo-MFP and Duet-MFP systems, the modules were secured on a fluidic plate that contained the microfluidic flow channels. The fluidic plate was placed atop the actuator plate that contained the electromagnetic actuators, and the two plates were separated by a Viton membrane. A fan-cooled heat sink was also added to the system to remove excess heat from the actuator plate. The actuator plate was connected to a computer and controller box via two ribbon cables. This controller box acted as the intermediate between the Quintet- MFP system and the PC running the custom LabVIEW pump programme.

**Figure 2:**
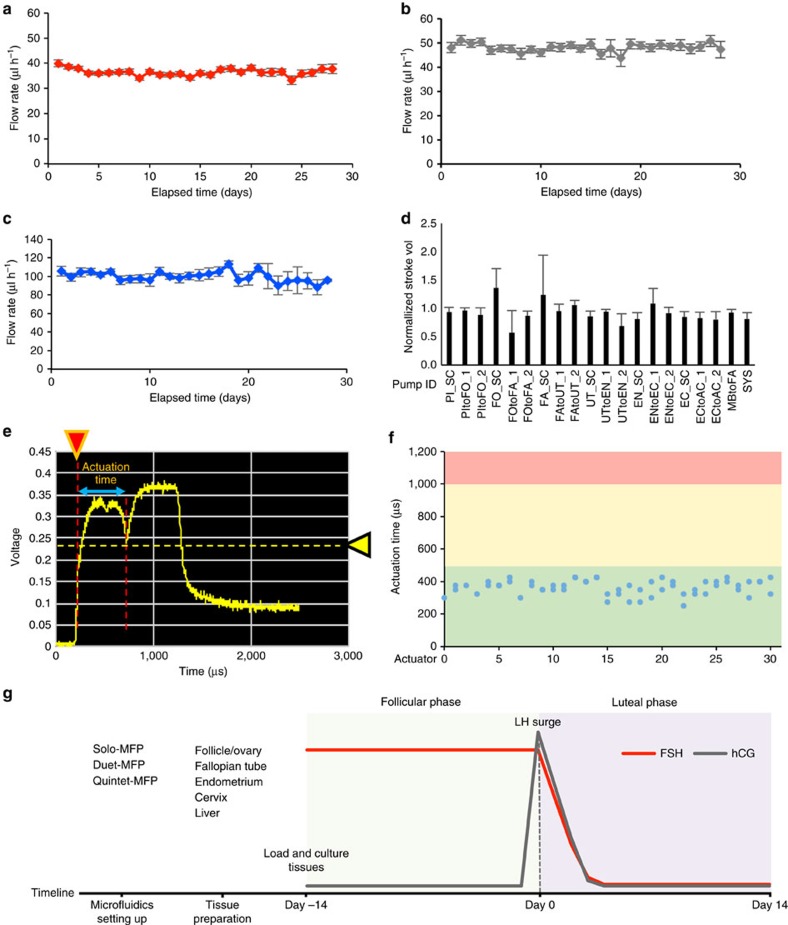
The pumping scheme of microfluidic platforms and experimental procedures. The average flow rates of (**a**) Solo-MFP (*n*=19), (**b**) Duet-MFP (*n*=23) and (**c**) Quintet-MFP (*n*=10) systems. (**d**) Average stroke volume for each pump pathway in the Quintet-MFP over the course of 33 days normalized with respect to the initial value. (**e**) Example oscilloscope reading illustrating how the actuation time was measured to determine the status of actuator function. Red arrow: trigger position; yellow arrow: trigger level. (**f**) A plot of Quintet-MFP actuator status at the beginning of an experiment with the upper and lower bounds of 625 and 250 μs, respectively. (**g**) Timeline for microfluidic platform setup, tissue preparation and culture. Graphs in **a**–**d** display average+s.d. *n*=4 replicates for the microfluidic platform testing.

For all Solo-MFP and Duet-MFP experiments, the empirically derived nominal through-system flow rate was set to 40 μl h^−1^ to create physiologic concentrations of oestradiol and progesterone as determined by mathematical modelling of system pharmacokinetics. The average through-system flow rates across 28 days is shown for each system in Fig. 2a,b. For Quintet-MFP experiments, the empirically derived through-system flow rate was set to 100 μl h^−1^ to create a physiologic profile of oestradiol and progesterone as well as to minimize the concentration lag between sequential modules (Supplementary Figs 1–3). The average through-system flow rates across 28 days for the Quintet-MFP are shown in Fig. 2c. The through-system flow rates for all three systems were found to consistently pump within close range of the target values for the entirety of 28-day experiments, and in the case of the Quintet-MFP, for periods of more than 100 days. In addition, the stroke volumes (that is, the fluid volume displaced in a single pump stroke) were found to exhibit little variation across all pump pathways during a month-long pump cycle (Fig. 2d).

The status of actuator function was periodically evaluated *in situ* by monitoring the voltage pulse using an oscilloscope (Fig. 2e). The actuation time is defined as the amount of time required to completely open the actuator after the application of current to the electromagnet of the actuator (Fig. 2f). From empirical data, actuation times within the range of 250–625 μs were considered indicative of long-term stability of mechanical function; actuation times below the lower limit indicated incomplete opening of the valve while actuation times longer than the upper limit indicated that the actuation distance may be too long for reliable actuation. *In situ* actuator monitoring together with daily flow measurements enabled consistent pumping over month-long studies. The timelines for these studies, including microfluidic platform setup and tissue preparation and culture, are shown in Fig. 2g.

### Microfluidic culture supported follicle development

**Figure 3:**
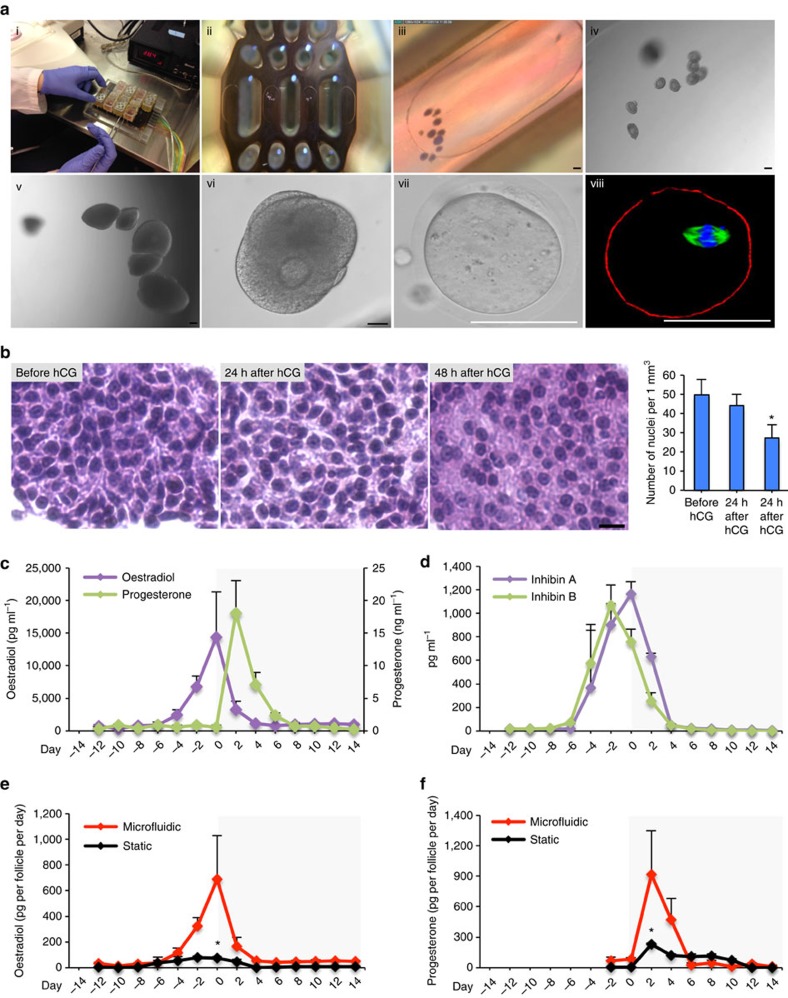
Microfluidic platform supported follicle maturation and hormone secretion in the Solo-MFP. (**a**) Multiple follicle culture in the Solo-MFP (stages i–iii); microfluidic culture supported follicle growth from primary stage to antral stage (iv–vi); and, following hCG stimulation, completion of the first meiotic division by the oocyte was achieved oocyte completed the first meiosis indicated by well-organized microtubule fibres (green), tightly aligned chromosomes (blue), and the round appearance of F-actin (red; stages vii–viii). (**b**) Granulosa cells showed similar morphological changes as seen *in vivo* following luteinization, indicated by hypertrophy and decreased nucleus to cytoplasm ratio. (**c**,**d**) Ovarian hormone secretions of oestradiol (E2) and progesterone (P4; **c**) and inhibin A and inhibin B (**d**) over 28 days of culture in the Solo-MFP. (**e**,**f**) Comparison of E2 (**e**) and P4 (**f**) secretion rates between microfluidic and static cultures. Graphs in **b**,**f** display average+s.d. *P<0.05 comparison of the number of nuclei per mm^3^ in follicles before and after hCG treatment (**b**), and hormone secretion rates between microfluidic and static cultures (**e**,**f**). Scale bar, 50 μm (**a**) and 10 μm (**b**). *n*=3–6 replicates for both the microfluidic and static culture.

*In vitro* follicle growth is a well-established model to study ovarian function^19^,^20^,^21^. To test whether the microfluidic system supports follicle growth, we initially performed experiments in Solo-MFP systems using primary mouse follicles (Fig. 3a). Mouse gonadal tissue from 12-day-old CD-1 mice was used throughout these studies because healthy ovaries are never removed from women, except under extraordinary circumstances such as in the case of a sterilizing cancer diagnosis^22^. As has been demonstrated previously, murine and human primary follicles behave similarly in static culture^21^. For our microfluidic cultures, follicle-stimulating hormone (FSH) was provided at a concentration of 10 mIU ml^−1^ through the first 14 days (day −14 to day 0) to mimic follicular phase gonadotropin levels (Fig. 2g). To phenocopy the luteinizing hormone (LH) surge, we created an algorithm called ‘surge-purge' that generated peak human chorionic gonadotropin (hCG) on day 0 (Fig. 2g). Hormones were then brought to baseline for the remaining days of culture (day 1 to day 14; Fig. 2g). All data are presented with the hCG surge as day 0 to permit comparison of follicular and luteal phase patterns to phases of the human menstrual cycle (Fig. 2g). Follicle architecture and the spatial relationship of germ cells and their supporting somatic cells were maintained in the Solo-MFP. Moreover, follicle growth was supported from primary/early secondary stage to antral stage (Fig. 3a). After hCG stimulation, follicles released metaphase II (MII) oocytes with barrel-shaped bipolar spindles and tightly aligned chromosomes, which was termed MFP ovulation (Fig. 3a). Once MFP ovulation occurred, the granulosa cells differentiated into luteal cells as indicated by the granulosa cell hypertrophy and the significantly reduced number of cell nuclei in a defined area (Fig. 3b, *P*<0.05 analysis of variance (ANOVA) with Tukey's range test). These results demonstrate that the microfluidic environment of the Solo-MFP was capable of supporting individual ovarian follicle growth, maturation, MFP ovulation, and granulosa cell luteinization.

### Twenty-eight-day hormone production in the Solo-MFP

Follicle hormone secretion was next examined within microfluidic culture. Following the establishment of microfluidic support and development of ovarian follicles, follicular hormone secretion was investigated. During the follicular phase, 17β-oestradiol (E2) production gradually increased and peaked on day 0 when follicles reached maturation (Fig. 3c). During the luteal phase, the progesterone (P4) concentrations were markedly increased and peaked two days after hCG treatment (Fig. 3c, *P*<0.05 ANOVA with Tukey's range test). Compared to steroid hormones secreted in static culture, follicles exposed to dynamic flow had significantly higher E2 and P4 production (pg per follicle per day; Fig. 3e,f, *P*<0.05 ANOVA with Tukey's range test). Next, we examined two peptide hormones, inhibin A and inhibin B, that are known to be secreted in the follicular phase to assess non-steroidal patterns of follicular function. Inhibin A, is a member of the TGFβ superfamily exhibited a secretion pattern similar to that of E2, peaking when follicles reached maturation and subsequently decreasing, remaining low during the luteal phase (Fig. 3d). The secretion of inhibin B gradually increased until day −2 of the follicular phase, then decreased and remained low in the later stage of the luteal phase (Fig. 3d). Taken together, these patterns of inhibin A and inhibin B, as well as E2 and P4, are consistent with healthy ovarian follicle performance^23^,^24^,^25^. These human-like patterns were entirely controlled by exogenous FSH and LH. These data show what has long been suspected; that pituitary hormones set the tempo of each reproductive cycle, both *in vivo* and now in microfluidics.

### Ovarian explant cultures in the Solo-MFP and Duet-MFP

**Figure 4:**
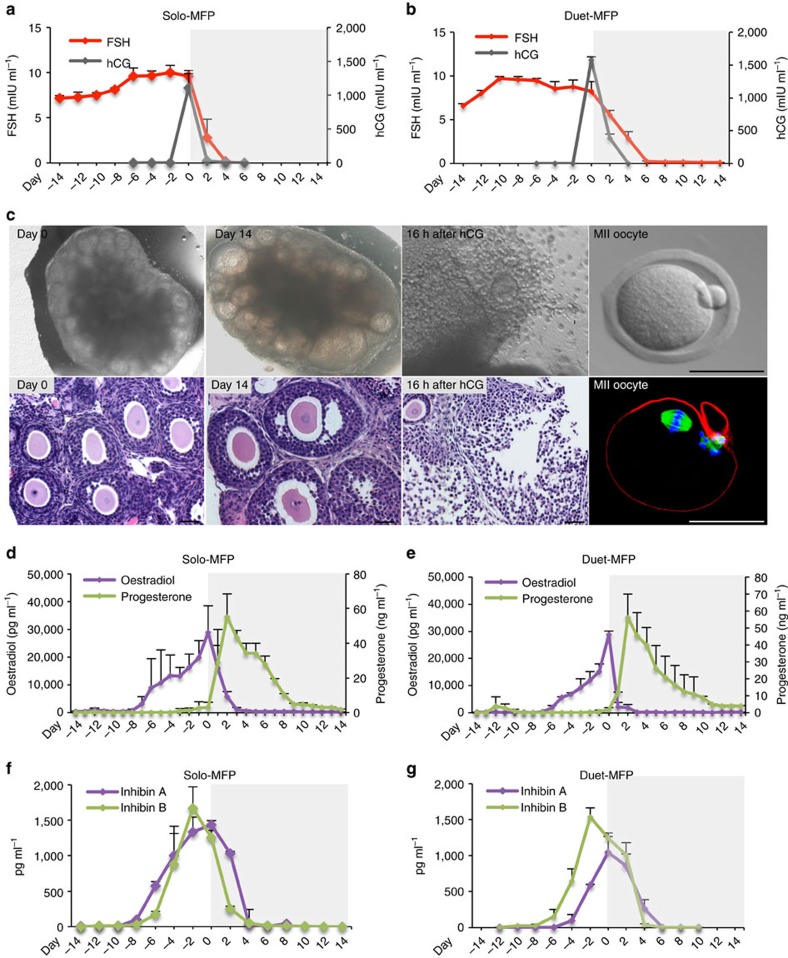
Ovarian explant cultures supported follicle development and hormone secretion in the Solo-MFP and Duet-MFP. (**a**,**b**) Concentrations of pituitary hormones, FSH and hCG, in the acceptor module during 28-day microfluidic culture. (**c**) Ovarian explants were cultured in the Solo-MFP and Duet-MFP, in which follicles developed from the pre-antral to antral stage, and extruded MII oocytes following hCG stimulation. (**d**,**e**) Secretion of oestradiol (E2) and progesterone (P4) in the Solo-MFP and Duet-MFP. (**f**,**g**) Secretion of inhibin A and inhibin B in the Solo-MFP and Duet-MFP. Graphs in **a**,**b**,**d**–**f** display average+s.d. Scale bar, 50 μm. *n*=3–5 replicates for the Solo-MFP and Duet-MFP microfluidic cultures.

Based on the success of follicle cultures in the Solo-MFP, we then used our microfluidic systems to culture ovarian explants. Ovaries were collected from 12-day-old CD-1 mice; these tissues included follicles from all classes (primordial, primary, and secondary). Ovaries were quartered and two pieces were placed in a single cell culture insert and cultured in either the Solo-MFP or Duet-MFP for 28 days. The treatment strategies for the pituitary hormones, FSH and hCG, were the same as those used in the follicle microfluidic cultures (Fig. 4a,b). Both the Solo-MFP and Duet-MFP supported follicle growth from primary/early secondary to multilayer and antral stages within the ovarian explants (Fig. 4c). After hCG stimulation, mature follicles displayed cumulus-oocyte complex expansion and released MII oocytes. Additionally, granulosa cells differentiated into luteal cells (Fig. 4c). Both steroid (E2 and P4) and peptide (inhibin A and B) hormones exhibited secretion patterns similar to those seen in the microfluidic follicle cultures (Fig. 4d–g), suggesting that ovarian tissue is a sufficient proxy for individual follicles in our systems, and that the Solo-MFP and Duet-MFP support each of the follicular endpoints described above.

### Integration of human tissues into the Quintet-MFP

**Figure 5:**
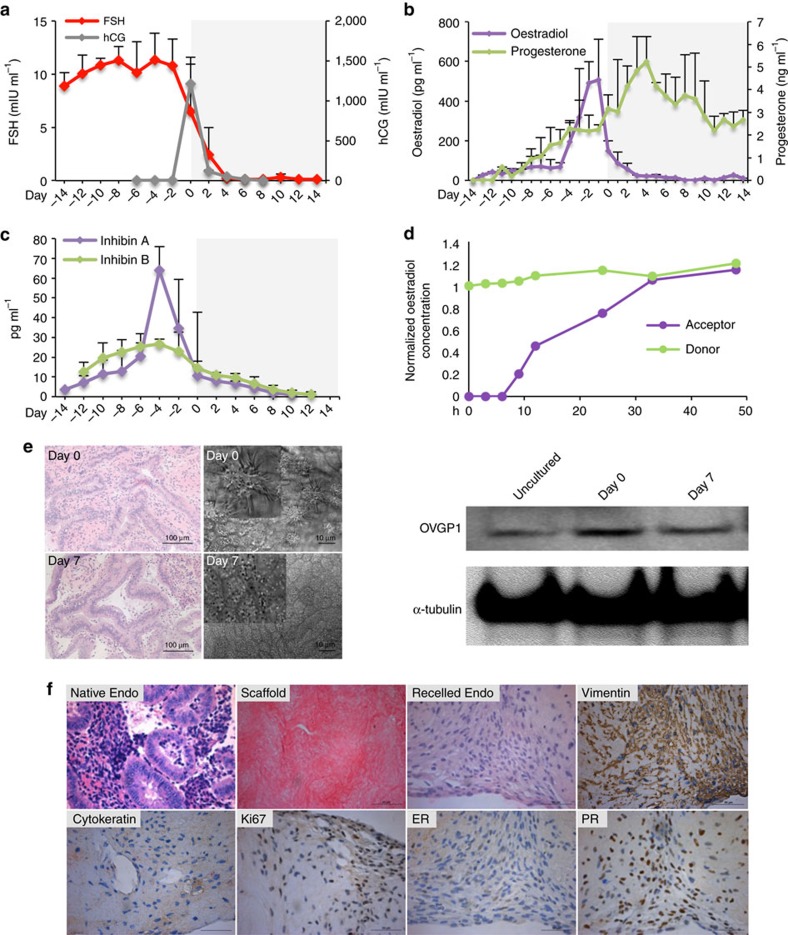
The Quintet-MFP supported the integration of female reproductive tissues. (**a**) Concentrations of pituitary hormones, FSH and hCG, during 28-day culture in the Quintet-MFP. (**b**,**c**) Ovarian hormone secretion during 28-day microfluidic culture with oestradiol (E2) and progesterone (P4) in **b** and inhibin A and inhibin B in **c**. (**d**) Dynamic E2 concentrations in the donor and acceptor modules over 48 h. (**e**) Images of fallopian epithelium histology, cilia image and OVGP1 expression on days 0 and 7 (14 and 21 days of microfluidic culture) in the Quintet-MFP. (**f**) Human endometrium tissue before and after decellularization, after recellularization, and immunohistochemistry staining of vimentin, cytokeratin, Ki67, ER and, PR on day 14 (28 days of microfluidic culture) in the Quintet-MFP. Graphs of **a**–**d** display average+s.d. Scale bar, 100 μm for fallopian epithelium histology, 10 μm for cilia images (**e**), and 50 μm for endometrium images (**f**). OVGP1 expression: α-tubulin as internal control. *n*=3 replicates of the integrated tissue culture in the Quintet-MFP.

To generate a hormonally coupled *ex vivo* female reproductive tract, we cultured murine ovary human fallopian tube, endometrium, ectocervix, and liver tissues for 28 days in the Quintet-MFP; this combination of tissues within the system was subsequently termed EVATAR. This system maintained the consistent pituitary hormone circulation and recapitulated the pituitary hormone control of relevant tissue function, effectively mimicking a 28-day human menstrual cycle (Fig. 5a). E2 and P4 peaked in the follicular and luteal phases, respectively (Fig. 5b). However, the absolute concentrations for both steroid and peptide hormones were significantly decreased compared to hormone concentrations in both the Solo-MFP and Duet-MFP (60-fold decreased for E2, 10-fold decrease for P4, 20-fold decrease for inhibin A, and 60-fold decrease for inhibin B, *P*<0.05 ANOVA with Tukey's range test; Fig. 5b,c). Since all materials used were the same between microfluidic platforms, and no hormone binding to the materials was observed (Fig. 5d), these data suggest that the integration of multiple tissues altered the upstream ovarian hormone expression patterns and/or that downstream reproductive tissues consumed ovarian hormone secretions.

### Microfluidic culture of human fallopian explants

A static co-culture system of murine follicles and human fallopian tissue has previously been established in our laboratory^26^. Our work confirmed that the cultured human fallopian tube epithelium, including ciliated and secretory cells, could be regulated by exogenous steroid hormones secreted by co-cultured ovarian tissues. Specifically, cilia length, cilia-beating status, and oviduct-specific glycoprotein 1 (OVGP1) secretion provided hallmarks of the functional changes in the human fallopian epithelium during the menstrual cycle^27^,^28^,^29^,^30^. In the current study, fallopian explants were incorporated into the Quintet-MFP to facilitate a more complex co-culture environment beyond simple static cultures. After 21 days of culture, tissue viability was confirmed by histology and cilia beating was still observed (Fig. 5e and Supplementary Videos 1 and 2). Moreover, the thickness of the fallopian epithelium on day 0, prior to hCG stimulation, was greater than that of tissues on day 7. Additionally, OVGP1 expression was higher on day 0, following follicular phase E2 stimulation, than on day 7 (Fig. 5e).

### Microfluidic culture of human endometrium

Human uterine endometrial tissue explants do not survive in culture long term. We developed a 3D culture system consisting of endometrial cells grown in decellularized uterine scaffolds, which were then placed in static and microfluidic cultures (Fig. 5f). Stromal and epithelial cells within the recellularized scaffolds were delineated by immunohistochemical staining of vimentin and cytokeratin, respectively (Fig. 5f). The stromal cells stained positively for Ki67, and expressed estrogen receptors (ER) and progesterone receptors (PR) at the end of the 28-day culture, indicative of active cell proliferation and uterine stromal cell characteristics (Fig. 5f).

### Microfluidic culture of human ectocervix explants

**Figure 6:**
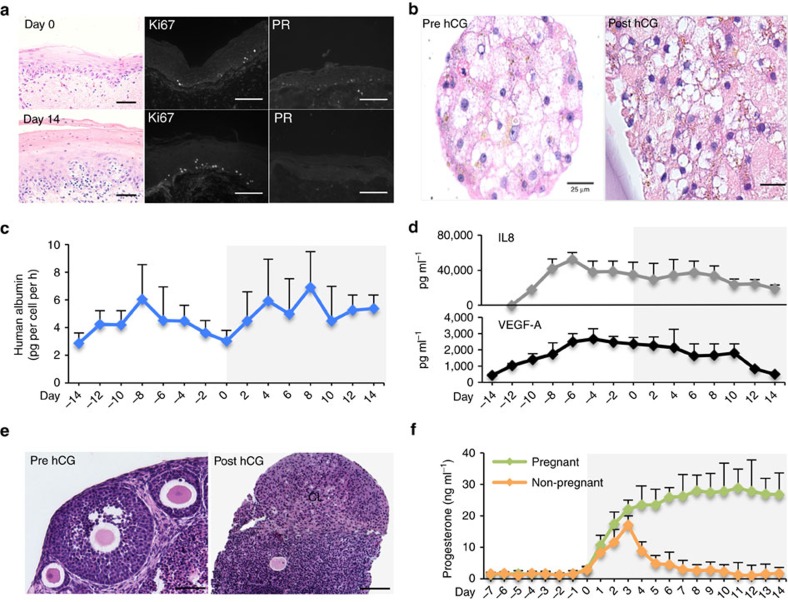
The Quintet-MFP supported female reproductive and non-reproductive tissues and pregnancy-like hormone condition. (**a**) Ectocervix histology, and Ki67 and PR staining at the end of follicular (day 0) and luteal (day 14) phases. (**b**) Histology of liver microtissues before (day −14) and after (day 14) hCG treatment. (**c**) Human albumin production over 28 days of microfluidic culture. (**d**) Production of IL8 and VEGF over 28 days of microfluidic culture. (**e**) Histology of ovarian tissue in the Quintet-MFP on day 0 (pre hCG) and day 8 (continuously cultured with hCG). (**f**) Ovarian progesterone secretion with and without hCG treatment. Graphs in **c**,**d**,**f** display average+s.d.'. Scale bar, 10 μm (**a**), 25 μm (**b**), and 100 μm (**e**). CL, corpus luteum; IL8, interleukin 8; VEGF-A vascular endothelial growth factor A. *n*=3 replicates of the integrated tissue culture in the Quintet-MFP.

To model hormone response in human ectocervix tissue *in vitro,* 3 mm biopsy punches of human ectocervix explants were cultured without hormones for 2–5 days before integration into the Quintet-MFP. Tissue was harvested at regular intervals throughout the entire 28-day cycle for histological analysis. The cultured ectocervix maintained its stratified squamous epithelial tissue architecture with proliferative potential as assessed by histology and Ki67 immunoreactivity, respectively (Fig. 6a). At the end of the follicular phase, when E2 concentrations peaked, the ectocervix explants prominently expressed PR in both the epithelium and stroma, while this receptor was undetectable at the end of the hormone cycle (day 14), when E2 concentrations decreased (Fig. 6a). These findings suggest that ectocervix tissue responded to E2 secreted from the upstream ovarian tissue in this microfluidic culture system.

### Microfluidic culture of human liver microtissues

Human liver microtissues were also included in the Quintet-MFP to explore non-reproductive tissue stability within our microfluidic culture system and because this organ will be an important tool in future metabolic studies. Over 28 days of microfluidic culture, liver microtissues retained their structure, as measured by the tight cellular contacts and nuclear staining before and after hCG stimulation (Fig. 6b). In addition, albumin secretion was stable throughout the 28-day culture period (Fig. 6c).

### Cytokine expression in microfluidic culture

A variety of other factors are made at constitutive levels by reproductive tract tissues that are not hormonally controlled. We examined two such cytokines, interleukin 8 (IL8) and vascular endothelial growth factor A (VEGF-A) in the Quintet-MFP. IL8 and VEGF-A peaked in the follicular phase and remained relatively constant through the end of the luteal phase (Fig. 6d), suggesting that the integrated system supports both reproductive and non-reproductive secretions throughout extended culture.

### Microfluidic culture supported pregnancy-like hormone control

As an alternative approach to studying reproductive tissues in our microfluidic systems, we asked whether we could prolong luteal phase function using the physiological cues associated with pregnancy in an attempt to prolong luteal phase function. Briefly, once ovulation occurs, the mature egg is released into the fallopian tube where, if sperm are available, it will be fertilized before moving into the uterus for implantation. The fertilized embryo and newly developing placenta produce and secrete hCG and prolactin at high levels, which rescues the corpus luteum (CL) to support an ongoing pregnancy^31^. We mimicked this ‘pregnancy'-like state and maintained the CL for the full 14 days of the luteal phase following MFP ovulation in the Quintet-MFP (Fig. 6e). Moreover, the ‘pregnant' luteal tissue produced significantly higher levels of P4 compared to the ‘non-pregnant' system (Fig. 6f, *P*<0.05 ANOVA with Tukey's range test). This experiment demonstrates the power of the integrated EVATAR in the Quintet-MPF to elucidate fundamental mechanisms of reproductive function, opening the door to new approaches to drug discovery and toxicological studies.

## Discussion

During each ovarian cycle, follicles produce steroid and peptide hormones to regulate downstream tissue functions, such as endometrial growth and menstruation^32^. However, there are few *in vivo* and *in vitro* models available to recapitulate a complete 28-day human menstrual cycle. By using our previously established *in vitro* culture methods in our microfluidic systems, ovarian tissues model *in vivo* human 28-day follicular and luteal phase hormone synthesis^23^,^33^,^34^, and provide female steroid and peptide hormones for downstream human fallopian, endometrium, ectocervix, and liver tissues. Compared to static follicle culture, the introduction of dynamic media flow promotes ovarian steroid hormone production by follicles. Ovarian tissue culture was then tested in both the Solo-MFP and Duet-MFP, and our results demonstrate that these systems supported follicle maturation and differentiation, and had ovarian hormone secretion profiles similar to those seen with isolated follicle culture methods. By altering culture conditions to include sustained hCG, ovarian tissues formed CLs and maintained high P4 production during the luteal phase, as seen during pregnancy.

In our previous study, it was difficult to sustain cilia beating beyond 7 days in static culture^26^; however, in the presence of dynamic flow in the Quintet-MFP, ciliary beating could be maintained for up to 21 days. Cilia growth and OVGP1 expression were induced by E2 and suppressed by P4, which was consistent with our previous findings in the follicle and fallopian co-culture system^26^. IL8 and VEGF-A have been reported to benefit CL function by maintaining high P4 production^35^,^36^. Our study showed that both IL8 and VEGF-A peaked around the later stage of the follicular phase and plateaued through the majority of the luteal phase, which may give rise to the different progesterone secretion patterns seen in the Solo/Duet-MFP and Quintet-MFP systems.

Our engineered endometrial scaffolds co-cultured with other female reproductive tissues survived 28 days in the Quintet-MFP, suggesting this system is capable of sustaining the recellularized scaffolds for extended culture in the presence of ovarian hormones, E2 and P4. The presence of ER and PR also suggests that the cells in the recellularized scaffold were hormone-responsive. In addition to endometrial cells, to date, there are limited *in vitro* systems that include other cell types, such as leukocytes and natural killer cells, which play active roles in the remodelling of the endometrium as well as influencing implantation and menstruation *in vivo*^37^,^38^,^39^. Our decellularized endometrial scaffold provides a native tissue-like environment and structure for cells to interact. in future years, circulating cells like leukocytes could be envisaged to move between wells with the aid of fluid flow.

After 28 days of culture in the Quintet-MFP, we verified that the ectocervix explants maintained fully differentiated squamous architecture characteristic of native ectocervix. As shown by Ki67 expression throughout the culture, the basal and parabasal layers of cells maintained proliferative potential in both the follicular and luteal phases. Moreover, PR expression was increased in response to the elevated E2 from upstream ovarian tissue culture.

Liver metabolism is an important component when considering pharmacological and toxicological testing. We demonstrate that liver microtissues survived the length of a 28-day female hormone cycle in the Quintet-MFP and maintained steady production of human albumin, which binds steroid hormones produced during microfluidic cultures. However, further studies are needed to investigate the xenobiotic metabolism of liver microtissues and whether the metabolic activity could be affected by sex hormones.

There are limited ways to effectively study whole tissues and tissue–tissue interactions. The female reproductive organs are especially dynamic, as they respond to fluctuating hormonal concentrations driven by the pituitary gland and ovary in preparation for ovulation, fertilization, embryo implantation, and placentation. Our work provides evidence that tissues of the female reproductive tract, as well as peripheral organs can be integrated into a microphysiologic, dynamic, and microfluidic culture system termed EVATAR. This powerful tool allows organ–organ integration of hormonal signals in a manner that phenocopies the human menstrual cycle and pregnancy. The Solo-MFP, Duet-MFP and Quintet-MFP were invented to investigate both single-tissue response and multi-tissue interactions in a manner that maintains the 3D architecture of each tissue. These systems produce highly controllable, stable flow patterns for around 100 days Because of the reconfigurable nature inherent in the design of these systems, the Solo-MFP, Duet-MFP and, Quintet-MFP can be used to investigate numerous combinations of tissue–tissue interactions beyond the female reproductive tract, opening up a brand new method for *in vitro* tissue culture that is expected to improve the pace and quality of across the spectrum of biological and pharmacological research.

## Methods

### Animals and human reproductive tissues

Ovaries and primary/early secondary follicles were harvested from 12-day-old CD-1 female mice. All mice were housed in polypropylene cages and provided food and water *ad libitum*. Animals were kept on a 12-h light/dark cycle (7:00 AM to 7:00 PM) at 23±1 °C with 30–50% relative humidity. Animals were fed Teklad Global irradiated 2,919 or 2,916 chow (Teklad Global), which does not contain soybean or alfalfa meal to minimize the exposure to phytoestrogens. All methods used in this study were approved by the Northwestern University Institutional Animal Care and Use Committee and correspond to the National Institutes of Health guidelines and public law. Human fallopian tube, uterine endometrium and ectocervical tissues were obtained from women undergoing routine salpingectomies and hysterectomies at Northwestern University Prentice Women's Hospital (Chicago, IL, USA). All experiments, procedures and methods were carried out in accordance with the IRB-approved guidelines and regulations. All female patients signed the informed consent form and were aware that their reproductive tissues would be used in research.

### Pharmacokinetic modelling of microfluidic platforms

A seven-compartment model was simulated to estimate the kinetics of distribution of oestradiol and other relevant factors. This model was used to set flow rates and volumes of modules within the system to create physiologic oestradiol concentrations and system response times of less than 1 day. The equations are shown below and were solved in Mathworks Matlab version R2012b.


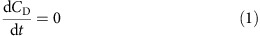



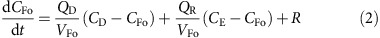



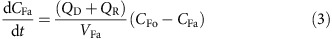



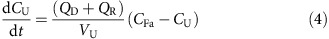



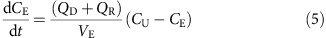



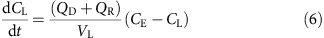



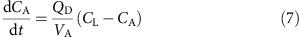


Where *C*_D_=concentration in Donor module (nmol l^−1^), *C*_Fo_=concentration in follicle module (nmol l^−1^), *C*_Fa_=concentration in fallopian module (nmol l^−1^), *C*_U_=concentration in uterine module (nmol l^−1^), *C*_E_=concentration in ectocervix module (nmol l^−1^), *C*_L_=concentration in liver module (nmol l^−1^), *C*_A_=concentration in acceptor module (nmol l^−1^), *V*_D_=volume in donor module (L), *V*_Fo_=volume in follicle module (L), *V*_Fa_=volume in fallopian module (L), *V*_U_=volume in uterine module (L), *V*_E_=volume in ectocervix module (L), *V*_L_=volume in liver module, *V*_A_=volume in acceptor module (L), *Q*_D_=flow rate from the mixer through the organ modules to the collection port (l min^−1^), *Q*_R_=recirculating flow rate from the ectocervix module to the follicle module (l min^−1^) and *R* is a production or elimination rate. The pharmacokinetic modelling data are shown in the Supplementary Information.

### Microfluidic culture setting up and procedures

Prior to insertion of microtissues into microfluidic culture systems, all systems were sterilized using a Sterrad systems (Advanced Sterilization Products). Microfluidic platforms were assembled with appropriate modules under sterile conditions. Media was sampled from the sampling module on a daily basis and the through-system flow rate was determined based on the media volume collected. Samples were immediately stored at −20 °C for later analysis. In the case of the Quintet-MFP, the actuation time of each actuator was periodically measured using an oscilloscope. For actuation times outside the range of 250–625 μs, the fluid path was diverted to alternate pump pathways in order to maintain desired flow rates.

### Ovary tissue collection and culture

Mouse ovaries and primary/early secondary follicles (100–120 μm) were isolated from 45 12-day-old CD-1 female mice. Animals were randomly used for all experiments. Only follicles that were morphologically intact were selected for encapsulation and culture. Follicles were placed in maintenance media containing 50% minimal essential medium (αMEM Glutamax, Sigma-Aldrich) and 50% Nutrient Mixture (F-12 with Glutamax, Sigma-Aldrich) with 1% fetal bovine serum (FBS, Life Technology) for 2 h before alginate encapsulation. Ten follicles were encapsulated by placing follicles in the centre of a 5 μl 0.5% alginate (NovaMatrix) drop on a polypropylene mesh and the alginate drops were immersed in the solution of 50 mM CaCl_2_ and 140 mM NaCl for 2 min to allow for crosslinking before transferring to the growth media^40^. Ovaries were cut into four even pieces within the dissection medium for ovarian explant microfluidic culture. Two quarters of ovarian pieces placed on a 0.4 μm cell culture insert (EMD Millipore Co), or four alginate beads, were seeded in each microfluidic culture module containing 700 μl growth media (detailed below).

In order to recapitulate human 28-day menstrual cycle hormone control, ovary and follicles were cultured over the course of 28 days, with the inclusion of both follicular phase and luteal phase hormones. During the follicular phase (day −14 to day 0), ovarian explant/encapsulated follicles were cultured at 37 °C in 5% CO_2_ for 14 days with growth media containing 10 mIU ml^−1^ recombinant FSH (from A. F. Parlow, National Hormone and Peptide Program, National Institute of Diabetes and Digestive and Kidney Diseases). Growth media consists of 50% αMEM Glutamax and 50% F-12 Glutamax supplemented with 3 mg ml^−1^ BSA (MP Biomedicals), 0.5 mg ml^−1^ bovine fetuin (Sigma-Aldrich), 5 μg ml^−1^ insulin, 5 μg ml^−1^ transferrin and 5 μg ml^−1^ selenium (Sigma-Aldrich). On day 0, cultured ovary/follicles were stimulated with maturation media for 16 h at 37 °C in 5% CO_2_ in air (50% αMEM and 50% F-12 with 10% FBS, 1.5 IU ml^−1^ hCG (Sigma-Aldrich), 10 ng ml^−1^ epidermal growth factor (BD Biosciences) and 10 mIU ml^−1^ FSH). Some oocytes were collected for inspection to determine the oocyte maturation. Oocytes were considered to progress to MII stage if a polar body was present after *in vitro* maturation with hCG. During luteal phase (day 0 to day 14), ovaries/follicles were cultured in growth media without FSH. Media was collected every 24 h from the acceptor module of microfluidic platform, and the flow rate and ovarian hormone production were monitored. Static cultures were performed in parallel under the same conditions for each culture with replacement of 50% of media volume with fresh media every 48 h, and the ovarian explant/encapsulated follicles were randomly distributed to microfluidic and static cultures for hormone production comparison.

### Preparation of tissues for microfluidic culture

The dissection media, growth media and maturation media used for ovarian tissue culture were used to maintain, isolate and culture human reproductive tissues and liver tissues. Tissues were washed twice with warm PBS containing 1% penicillin/streptomycin twice, and then transferred into warm dissection medium. All human reproductive tissues were received and processed within 24 h of surgery. The human fallopian tube tissue was cut open, and the inner layer was mechanically dissected using forceps. This layer, primarily consisting of epithelium and underlying stroma, was cut into 2 × 2 mm pieces, placed on the 0.4 μm cell culture insert (EMD Millipore Co) and transferred to the growth media for culture. To obtain decellularized uterine endometrium scaffolds, patient-derived tissue was treated with a combination of sodium deoxycholate and Triton X-100, followed by nuclear digestion and extensive rinsing in growth media. The decellularized endometrium scaffolds were stored in PBS containing 1% penicillin/streptomycin at 4 °C. Received human endometrial tissue was digested with collagenase, and the resulting stromal and epithelial cells were cultured on two-dimensional (2D) plates without passaging for 7days. A thick suspension of endometrial cells was seeded on top of decellularized endometrial scaffolds within the cell culture insert, and the recellularization was allowed to occur over 8 weeks in growth media before being integrated with other tissues. Received human ectocervix tissues were trimmed of excess stroma until pieces were ∼1 mm thick. Three-millimetre biopsy punches of ectocervix pieces were transferred to the cell culture insert for culture. The ectocervix tissues were cultured without hormones for 2–5 days before co-cultured with other tissues.

3D InSight Human Liver Microtissues (InSphero AG) were generated from primary human hepatocytes (1,000 hepatocytes per microtissue) and liver non-parenchymal cells, including Kupffer cells, sinusoidal endothelial cells and hepatic stellate cells, were assembled to retain close cellular contacts and maintain liver-specific function that most closely resembles *in vivo* tissue^41^. The liver microtissues were encapsulated in 1% alginate hydrogels or loaded onto 3D-printed gelatin scaffolds with 10 microtissues per alginate bead or scaffold. The gelatin scaffolds were printed on glass slides as seven-layer thick, 15 × 15 mm squares, with 60° advancing angles between each layer, which created a 450–500 μm pore size. Four alginate beads or scaffolds with liver microtissues were placed on one cell culture insert.

To recapitulate pregnant-like hormone conditions in Quintet-MFP, ovarian tissues were continuously cultured with maturation media with an addition of 25 ng ml^−1^ prolactin (Sigma-Aldrich) during the luteal phase (day 0 to day 14).

### Histology

Follicles and liver microtissues cultured *in vitro* were fixed for 4 h at 4 °C in 3.8% paraformaldehyde in PBS. Ovary, fallopian tube, endometrium and ectocervix were fixed in 10% formalin overnight. Tissues were dehydrated in ascending concentrations of ethanol (50–100%) before being embedded in paraffin using an automated tissue processor (Leica). Serial 5 μm sections were cut for haematoxylin and eosin, immunofluorescence and immunohistochemistry staining.

### Immunofluorescence and immunohistochemistry

For oocyte spindle morphology and chromosome alignment analysis, gametes obtained following oocyte maturation were fixed in 3.8% paraformaldehyde containing 0.1% Triton X-100 (Sigma-Aldrich) for 1 h at 37 °C. Oocytes were washed three times in blocking solution with PBS containing 0.3% bovine serum albumin and 0.01% Tween-20, incubated overnight in a 1:50 dilution of mouse anti-α-tubulin (Cell Signaling Technology) in blocking solution. Then, oocytes were washed three times with blocking solution, mounted using Vectashield containing DAPI (Vector Laboratories) and analysed using an EVOS FL AUTO microscope (Life Technology) in a blinded manner. For sectioned tissues, antigen retrieval was performed using sodium citrate buffer (pH 6) in a pressure cooker for 35 min. Slides were cooled down for another 25 min before blocking. Sections were blocked with 10% goat serum for 1 h, before being incubated with primary antibodies. The endometrium was incubated in antibodies against Ki67 (1:100, Abcam), vimentin (1:400, Abcam), cytokeratin (1:200, Abcam), ER (1:75, Abcam) or PR (1:100, Dako), and the ectocervix was incubated with antibodies against PR (1:50, Cell Signaling) or ki67 (1:50, Abcam) overnight at 4 °C. After washing in PBS, sections were incubated with fluorescent secondary antibodies (1:500; Invitrogen) for 1 h, and visualized and imaged using Nikon E600 fluorescent microscope.

### Immunoblot analysis

Cultured human fallopian tissue was homogenized in ice-cold lysis buffer (20 mM Tris-HCl, pH 8.0, 137 mM NaCl, 10% glycerol, 1% NP-40, 2 mM EDTA) with proteinase inhibitor. Equal amounts of protein measured by the BCA protein assay kit (Thermo Scientific) were loaded into precast NuPAGE 4–12% gradient Bis-Tris. Gel electrophoresis was performed and proteins were dry-transferred using an iBlot system (Invitrogen) to a nitrocellulose membrane (Life Technologies). The blots were probed with a polyclonal anti-OVGP1 (1:1,000) antibody (LsBio) overnight at 4 °C, followed by anti-rabbit secondary antibody conjugated to horseradish peroxidase (1:5,000; Zymed). Proteins were detected by enhanced chemiluminescence primer (GE HealthCare Life Sciences) and exposed using a FluorChem HD machine (Alpha Innotech). The same blot was stripped with buffer (Thermo Scientific) and re-probed with a monoclonal anti-α-tubulin antibody (1:10,000; Sigma-Aldrich), followed by an anti-mouse secondary antibody conjugated to horseradish peroxidase.

### Monitoring of fallopian epithelial cilia beating

The fallopian tissues cultured in the Quintet-MFP were removed in order to monitor cilia beating after 14 and 21 days of microfluidic culture. Movies of cilia beating were acquired using an Andor Spinning Disk Confocal (Nikon) with 10 ms exposure time and 10 ms readout time. Each movie was taken in 50 individual frames. The wide field images of the fallopian tissue were acquired using an Andor Spinning Disk Confocal with a × 100 oil objective lens.

### Immunoassays

E2 and P4 were measured using commercial ELISA kits (E2: ALPCO; P4: IBL), and researchers were blinded to the animal group allocation when they performed ELISA to detect hormone production between microfluidic and static cultures. The assay validation protocol included spiking blank media with various concentrations of steroid (E2 or P4 reference preparations; Sigma-Aldrich) across the assay range to determine recovery and parallelism to the assay kit standard curve. Each assay included a blank media standard curve, which was used to calculate results. The limit of quantitation (functional sensitivity) was defined as the lowest concentration for which accuracy is within 20% of expected values and the intra-assay coefficient of variation (%CV) is less than 20%, and was determined by serial dilutions of a defined sample pool. For E2, intra-assay and inter-assay %CVs were 6.3% and 8.1%, respectively; functional sensitivity was 10 pg ml^−1^. For P4, intra-assay and inter-assay %CVs were 4.4% and 7.8%, respectively; functional sensitivity was 0.15 ng ml^−1^. Ovarian hormones (inhibin A and inhibin B) were measured using ELISA kits provided by Ansh Labs (Webster). Intra-assay, inter-assay CVs and functional sensitivity for inhibin A were 5.1%, 7.3% and 12 pg ml^−1^, respectively; 4.5%, 6.1% and 14 pg ml^−1^, respectively, for inhibin B; and 5.6%, 6.9% and 0.08 ng ml^−1^, respectively, for activin A; 6.9%, 8.1% and 0.34 ng ml^−1^, respectively, for AMH. IL8, VEGF (R&D Systems) and Albumin (Abcam) were measured using ELISAs. Albumin concentrations were normalized with the respect to the initial hepatocyte cell number and time. Intra-assay and inter-assay CVs and functional sensitivity for IL8 were 3.0%, 6.2% and 31.2 pg ml^−1^, respectively; 3.1%, 5.2% and 7.8 pg ml^−1^, respectively, for VEGF; 4.8%, 7.3% and 0.4 μg ml^−1^, respectively,for albumin. FSH and hCG were measured on the Siemens Immulite 2,000 automated chemiluminescent immunoassay analyser. Intra-assay and inter-assay CVs and functional sensitivity was 3.2%, 4.9% and 0.1 mIU ml^−1^, respectively, for FSH; 3.1%, 4.4% and 0.5 ng ml^−1^, respectively, for prolactin; 3.3%, 6.6% and 1.0 mIU ml^−1^, respectively, for hCG; and 2.7%, 5.2% and 2.0 nmol l^−1^, respectively, for SHBG.

### Dynamic E2 levels in the microfluidic system

To test whether the tissue secreted hormones bound to the microfluidic materials, growth media with 15,000 pg ml^−1^ E2 was added to the donor modules in Quintet-MFP, and media was collected from both donor and acceptor modules every 1–8 h. E2 concentrations were measured as described above.

### Statistical analyses

Hormone and protein secretion were analysed from three to five independent static cultures, Solo-MFP, Duet-MFP and Quintet-MFP microfluidic cultures. Data were analysed using one-way ANOVA, followed by Tukey range test for significant difference. Categorical data were analysed by the *χ*^2^-test; if significance was observed between groups, then we applied the Fisher's exact test. The significance level was set at *P*<0.05.

### Data availability

The authors declare that all data supporting the findings of this study are available within the article and its Supplementary Information Files or from the corresponding author upon reasonable request.

## Additional information

**How to cite this article:** Xiao, S. *et al*. A microfluidic culture model of the human reproductive tract and 28-day menstrual cycle. *Nat. Commun.* **8,** 14584 doi: 10.1038/ncomms14584 (2017).

**Publisher's note:** Springer Nature remains neutral with regard to jurisdictional claims in published maps and institutional affiliations.

## Supplementary Material

Supplementary Information Supplementary Figures and Supplementary Table.

Supplementary Movie 1 Fallopian tube cilia beating video after fallopian epithelium was cultured in Quintet- MFPTM for 14 days.

Supplementary Movie 2 Fallopian tube cilia beating video after fallopian epithelium was cultured in Quintet- MFPTM for 21 days.

Peer Review
