# Supplementary material for: A microfluidic culture model of the human reproductive tract and 28-day menstrual cycle
Source: Nat Commun. 2017 Mar 28;8:14584. doi: 10.1038/ncomms14584 (PMC5379057; doi:10.1038/ncomms14584)
Supplement: Supplementary Information — Supplementary Figures and Supplementary Table. [file ncomms14584-s1.pdf]

Supplementary Figure 1 showed the response time of the system to a step change in concentration in the Donor module. The simulation assumes $Q_D = Q_R$ = 100 µl/h, organ module volumes are 225 µl, and the sampling port volume is 200 µl. The calculated time required to reach 90% of the steady value in the sampling port is 1.03 Days. Supplementary Figure 2 shows the same system with Q_R = 0 µl/h. Note the system response time is slightly faster (T_{90} = 0.86 days), but there is a significant lag in concentration between modules. Finally, Supplementary Figure 3 showed the simulated estradiol concentration in each module when the estradiol production rate in the follicle module is set to an approximation of the human female serum estradiol concentration (adapted from dissertation “Modeling Endocrine Regulation of the Menstrual Cycle Using Delay Differential Equations”, Leona A. Harris, Department of Mathematics and Statistics, The College of New Jersey, Ewing, NJ 08628).

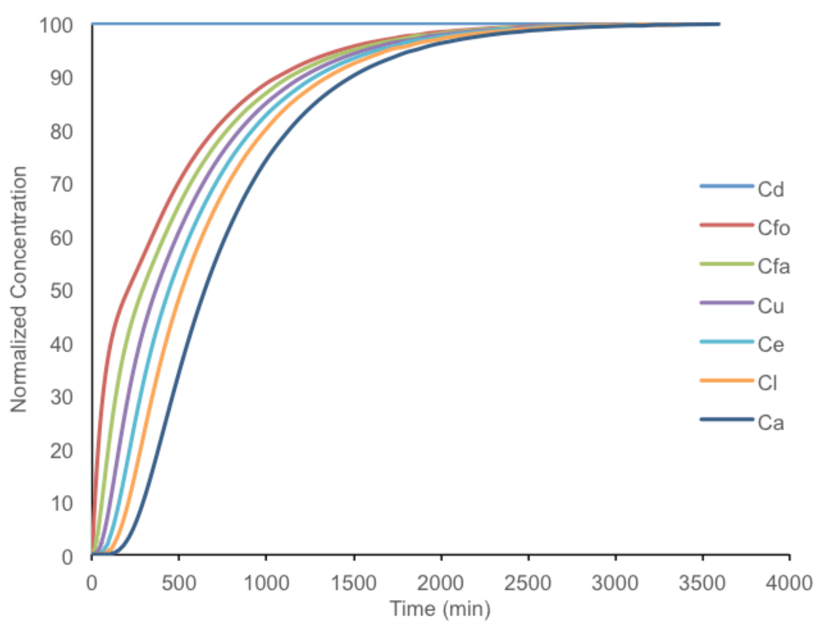

Supplementary Figure 1. System response profile towards $Q_D = Q_R = 100$ µl/h. MPS module concentration time profile resulting from a step change in concentration in Donor module from 0 to 100 (normalized concentration), Q_D = Q_R =100 µl/h.

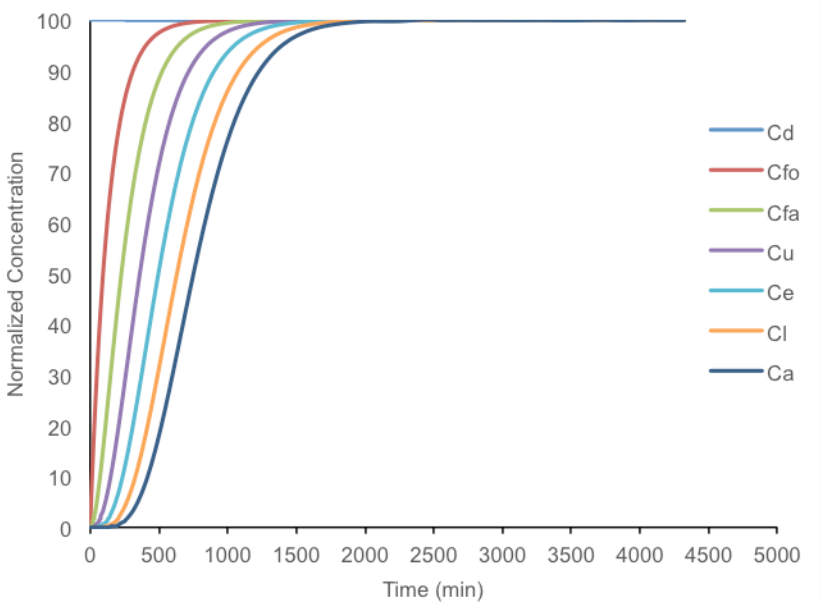

Supplementary Figure 2. System response profile towards Q_D=100 µl/h and Q_R = 0 µl/h. MPS module concentration time profile resulting from a step change in concentration in Donor module from 0 to 100 (normalized concentration), Q_D = 100 µl/h, Q_R = 0 µl/h.

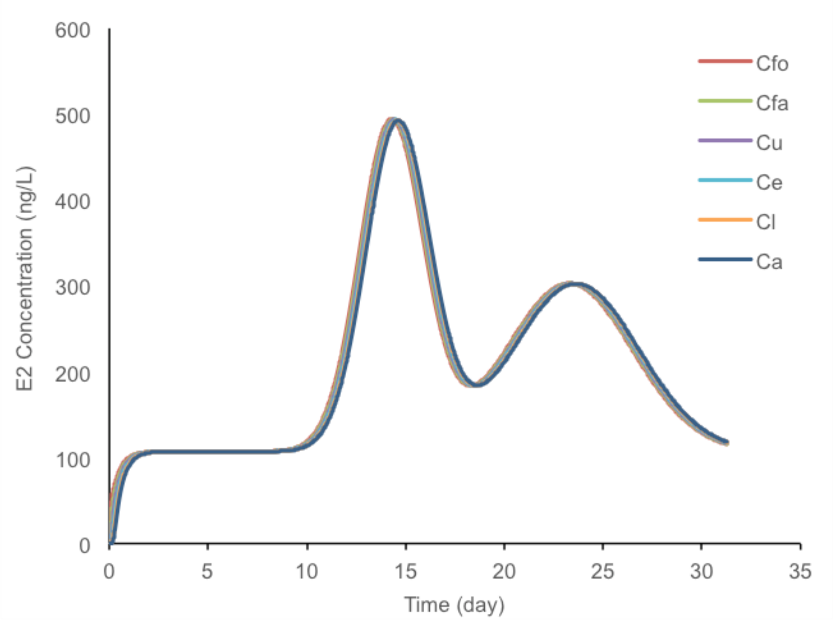

Supplementary Figure 3. System response profile towards dynamic estradiol stimulation. MPS module concentration time profile resulting from an approximation to a human physiologic serum estradiol concentration time profile, $Q_D = Q_R = 100$ µl/h.

Supplementary Table 1. Nomenclature and description of microfluidic technology and biological cultures.

Microfluidic Platform	Platform characteristics	Tissue Cultured	Biological Nomenclature
Solo-MFP™	Individual tissues	Mouse follicles	FemKube
	Unidirectional (40 μl/h)	Mouse ovaries	OviKube
		Human fallopian tubes	TubeKube
		Human uterus	UteroKube
		Human Cervix	CerviKube
		Human Liver	LiverKube
Duet- MFP™	Two Interacting tissues	Ovary	
	Unidirectional (40 μl/h)	Ovary + Fallopian	
		Ovary + Uterus	
		Ovary + Cervix	
Quintet-MFP™	Five Interacting tissues	All tissues above	EVATAR
	Recirculating (100 μl/h)		
