# Supplementary material for: A microfluidic culture model of the human reproductive tract and 28-day menstrual cycle
Source: Nat Commun. 2017 Mar 28;8:14584. doi: 10.1038/ncomms14584 (PMC5379057; doi:10.1038/ncomms14584)
Supplement: Peer Review [file ncomms14584-s4.pdf]

Reviewer #1 (Remarks to the Author)

This manuscript presents an engineering system designed to culture and maintain living tissue from female reproductive organs. The modular capacity, reconfigurability, and precise flow control demonstrated in this study are impressive and offer compelling advantages over traditional techniques for modeling the hormonal environment and multi-organ interactions in the reproductive system. The capability for long-term culture is another strength that makes this system an ideal platform for studying lengthy, periodic physiological processes such as the menstrual cycle. Undoubtedly the cell culture technology described in the manuscript is novel and has the potential for broad impact in reproductive medicine and related areas.

This paper, however, also raises several major concerns. First of all, the introduction of the manuscript fails to provide the rationale for this study, making it difficult for non-experts to understand the significance and potential of the developed technology. The authors discuss the current challenges in cell culture and drug development, but this discussion does not flow as a coherent story and focuses only on general problems that could be applied to any microphysiological system. The introduction should be re-rewritten to provide more context-specific discussion and explain the problems and limitations of conventional approaches in reproduction research that justify the development of microphysiological cell culture platforms.

On a related note, the authors should consider re-writing the entire manuscript to improve readability and re-organize the sections in a manner that is easier to follow. A lack of discussion on the rationale and significance of each set of experiments is a major drawback that obscures the impact of this work and is also detrimental to the flow of the manuscript. In addition, many of the studies detailed in the results section are purely phenomenological, failing to provide mechanistic insights into the important findings made in the microfluidic culture system. For example, the authors report that the production of steroid hormones in Solo-MFP was significantly higher than that in static culture. This is an interesting observation suggesting the benefit of dynamic culture environment but in-depth discussion on possible contributing factors is nowhere to be found in the manuscript. Considerable work is needed to improve the organization of the main body of the paper.

Another concern arises from a lack of control data. Comparison between MFP and static models is presented for culture of follicles but similar control studies should be conducted for some of the other tissue groups to convincingly show the advantage of MFP over conventional static culture. In some cases (e.g., liver module), model characterization is based on rudimentary biological analysis of a few readouts that are not entirely representative of in vivo phenotype. This may present challenges to morphological and functional validation of the model.

Finally it is highly questionable whether this study truly demonstrates the potential of the MFP platform to model and study multi-organ interactions. This is an essential question that remains unanswered in the current manuscript. The presented data seem very preliminary without any mechanistic information and raise more questions than answers; the authors provide only one sentence at the end of the paragraph to suggest multi-organ interactions that occur in this model but this conclusion is completely unsubstantiated. More rigorous studies are needed to validate the model and demonstrate/analyze cross-talk between different tissue types. Related questions that should be further investigated in this study are: what is the significance of including the endometrium and fallopian tube modules in the multi-organ system? Given that the liver model is integrated in the loop, is it possible to demonstrate the interaction between the liver and reproductive tissue using, for example, drug metabolites as suggested by the authors?

Reviewer #2 (Remarks to the Author)

This is a very interesting manuscript dealing with ex-vivo endocrine loops between organ modules for the ovary, fallopian tube, uterus, cervix and liver using a new and auspicious microfluidic

platform termed EVATAR. This innovative system which is supported by NIH and NCATS is intended to represent a phenocopy of the menstrual cycle and pregnancy.
Main advantage of this 3-D- chip platform with different organs is the possibility to look for long term dynamic interactions and to test the efficacy and toxicity of new drugs in vitro.
As described in detail in the manuscript, the microfluidic system supports the growth and maturation of murine follicles very well, and metaphase II oocytes are released after hCG stimulation. The follicles secrete E2 and P4 in a cyclic manner mimicking the menstrual cycle. Compared to static cultures E2 and P4 cyclic secretion rates of follicles are much higher in dynamic flow.
An important issue is the interaction of human reproductive tract tissues with the follicle cultures. The cultured human fallopian tube epithelium could be shown to be regulated by steroid hormones secreted by co-cultured ovarian tissues. This has recently been shown before by static co-cultures and published in "Molecular Human Reproduction" by Zhu et al., "Human fallopian tube epithelium co-culture with murine ovarian follicles reveals crosstalk in the reproductive cycle" (advance access published September 1, 2016) and should be cited.
In addition, the Paper of Arslan et al, Woodruff and Kim, Endocrinology, 156 (4): 1602-1609, 2015 about the 3D endocervix cultures in the presence of fluctuating levels of E2 and P4 mimicking the 28-day menstrual cycle should be mentioned as well.
Unfortunately, the co-culture of human endometrial tissue explants was not possible. The stromal and epithelial cells obtained by digestion of human endometrial tissue have been seeded on decellularized endometrial scaffolds. Finally, mainly stromal cells remained as shown by positive Vimentin reaction.
The authors could show that these systems produce highly- controllable, stable flow patterns for up to 105 days and that the cyclic secretion of steroid hormones can be mimicked in vitro. By using an interconnected platform, other human reproductive tract tissues could be influenced and regulated. Although for other tissues, besides human fallopian tube, the 3-D- cultures should be improved, this paper is important to scientists in this specific field and should be published in Nature Communications.

Reviewer #3 (Remarks to the Author)

The current manuscript has investigated the potential of microfluidic culture systems to mimic the menstrual cycle hormone control of different tissues within the female reproductive tract.

They demonstrate that external stimulation of murine follicles or ovarian biopsy (with FSH and hCG) can not only induce follicular maturation but also the appropriate estrogen and progesterone profile required for endometriual growth and maturation. This model system has potential to investigate alterations in hormonal influences on different aspects of reproductive tract biology at different parts of the cycle within a system that takes into account the interconnected organs of the reproductive tract and their influence on each other.

In general over the 28 days in culture tissue integrity was good, with the exception of endometrium which required reseeding of stromal cells and epithelial cells into cell free endometrial scafolds. However, the endometrial stroma contains a large number of leucocytes, numbers of which change dramatically thoughout the menstrual cycle, particularly in the mid-late secretory phase of the cycle. The exact role of these cells in endometrial biology are not known but they are proposed to play roles in implantation, menstruation, endometrial maturation etc. I accept that endometrial tissue does not do well in culture, even under near constant flow conditions. However, the absence of leucocytes in this experimental system needs to at least be commented on in the discussion as a potential limitation.

In addition, it was not clear if glandular structures were reproduced in the reconstituted endometrium and whether they became functionally active.

Response to reviewers' comments from previous submission:

The authors thank the referees for the thoughtful review of our manuscript and the positive comments. In particular, we are gratified that the reviewers appreciate the value of this contribution: "*Undoubtedly the cell culture technology described in the manuscript is novel and has the potential for broad impact in reproductive medicine and related areas"*.

Reviewer #1:

This manuscript presents an engineering system designed to culture and maintain living tissue from female reproductive organs. The modular capacity, reconfigurability, and precise flow control demonstrated in this study are impressive and offer compelling advantages over traditional techniques for modeling the hormonal environment and multi-organ interactions in the reproductive system. The capability for long-term culture is another strength that makes this system an ideal platform for studying lengthy, periodic physiological processes such as the menstrual cycle. Undoubtedly the cell culture technology described in the manuscript is novel and has the potential for broad impact in reproductive medicine and related areas.

Thank you for your comments on our study.

This paper, however, also raises several major concerns. First of all, the introduction of the manuscript fails to provide the rationale for this study, making it difficult for non-experts to understand the significance and potential of the developed technology. The authors discuss the current challenges in cell culture and drug development, but this discussion does not flow as a coherent story and focuses only on general problems that could be applied to any microphysiological system. The introduction should be re-rewritten to provide more context-specific discussion and explain the problems and limitations of conventional approaches in reproduction research that justify the development of microphysiological cell culture platforms.

We have rewritten the "introduction" in the revised manuscript, and included specific information about the female reproductive tract and the need for an *in vitro* model of this integrated organ system.

On a related note, the authors should consider re-writing the entire manuscript to improve readability and re-organize the sections in a manner that is easier to follow. A lack of discussion on the rationale and significance of each set of experiments is a major drawback that obscures the

impact of this work and is also detrimental to the flow of the manuscript. In addition, many of the studies detailed in the results section are purely phenomenological, failing to provide mechanistic insights into the important findings made in the microfluidic culture system. For example, the authors report that the production of steroid hormones in Solo-MFP was significantly higher than that in static culture. This is an interesting observation suggesting the benefit of dynamic culture environment but in-depth discussion on possible contributing factors is nowhere to be found in the manuscript. Considerable work is needed to improve the organization of the main body of the paper. Another concern arises from a lack of control data. Comparison between MFP and static models is presented for culture of follicles but similar control studies should be conducted for some of the other tissue groups to convincingly show the advantage of MFP over conventional static culture. In some cases (e.g., liver module), model characterization is based on rudimentary biological analysis of a few readouts that are not entirely representative of in vivo phenotype. This may present challenges to morphological and functional validation of the model.

We thank the reviewer for this helpful suggestion. We have included more specific rationale and significance statement for each element of the study in both Results and Discussion of the revised manuscript. Regarding to the higher expression of steroid hormones in microfluidic culture than that in static culture, we discussed that the dynamitic and uni-directional media flow patterns contribute to the promoted ovarian steroid hormone production. Overall, our goal is not to primarily compare the static to the microfluidic. Indeed, both static and dynamic fluid interactions have their utility. Rather, our goal was to invent an entirely new technology that could fundamentally enable physiologically relevant studies that cannot be accomplished in current static culture models, including the ability to provide changing levels of hormones in real time.

Finally it is highly questionable whether this study truly demonstrates the potential of the MFP platform to model and study multi-organ interactions. This is an essential question that remains unanswered in the current manuscript. The presented data seem very preliminary without any mechanistic information and raise more questions than answers; the authors provide only one sentence at the end of the paragraph to suggest multi-organ interactions that occur in this model but this conclusion is completely unsubstantiated. More rigorous studies are needed to validate the model and demonstrate/analyze cross-talk between different tissue types. Related questions that should be further investigated in this study are: what is the significance of including the endometrium and fallopian tube modules in the multi-organ system? Given that the liver model is integrated in the loop, is it possible to demonstrate the interaction between the liver and reproductive tissue using, for example, drug metabolites as suggested by the authors?

Thank you for pointing this out. We have included more discussion about the tissue-tissue interaction and cross-talk in the Quintet-MFP^{TM} microfluidic culture in the revised manuscript.

Reviewer #2:

This is a very interesting manuscript dealing with ex-vivo endocrine loops between organ modules for the ovary, fallopian tube, uterus, cervix and liver using a new and auspicious microfluidic platform termed EVATAR. This innovative system which is supported by NIH and NCATS is intended to represent a phenocopy of the menstrual cycle and pregnancy.

Main advantage of this 3-D- chip platform with different organs is the possibility to look for long term dynamic interactions and to test the efficacy and toxicity of new drugs in vitro.

As described in detail in the manuscript, the microfluidic system supports the growth and maturation of murine follicles very well, and metaphase II oocytes are released after hCG stimulation. The

follicles secrete E2 and P4 in a cyclic manner mimicking the menstrual cycle. Compared to static cultures E2 and P4 cyclic secretion rates of follicles are much higher in dynamic flow.
An important issue is the interaction of human reproductive tract tissues with the follicle cultures. The cultured human fallopian tube epithelium could be shown to be regulated by steroid hormones secreted by co-cultured ovarian tissues. This has recently been shown before by static co-cultures and published in “Molecular Human Reproduction” by Zhu et al., “Human fallopian tube epithelium co-culture with murine ovarian follicles reveals crosstalk in the reproductive cycle” (advance access published September 1, 2016) and should be cited.
In addition, the Paper of Arslan et al, Woodruff and Kim, Endocrinology, 156 (4): 1602-1609, 2015 about the 3D endocervix cultures in the presence of fluctuating levels of E2 and P4 mimicking the 28-day menstrual cycle should be mentioned as well.
Unfortunately, the co-culture of human endometrial tissue explants was not possible. The stromal and epithelial cells obtained by digestion of human endometrial tissue have been seeded on decellularized endometrial scaffolds. Finally, mainly stromal cells remained as shown by positive Vimentin reaction.
The authors could show that these systems produce highly- controllable, stable flow patterns for up to 105 days and that the cyclic secretion of steroid hormones can be mimicked in vitro. By using an interconnected platform, other human reproductive tract tissues could be influenced and regulated. Although for other tissues, besides human fallopian tube, the 3-D- cultures should be improved, this paper is important to scientists in this specific field and should be published in Nature Communications.

We thank reviewer #2 for the positive review of our work. We have included the additional citations that are mentioned, and they are references 15, 16, 17, 18, and 26 in the revised manuscript.

15. Arslan, S.Y. et al. Novel three dimensional human endocervix cultures respond to 28-day hormone treatment. *Endocrinology* **156**, 1602-1609 (2015).
16. Eddie, S.L. et al. Three-dimensional modeling of the human fallopian tube fimbriae. *Gynecologic oncology* **136**, 348-354 (2015).
17. Eddie, S.L., Kim, J.J., Woodruff, T.K. & Burdette, J.E. Microphysiological modeling of the reproductive tract: a fertile endeavor. *Experimental biology and medicine* **239**, 1192-1202 (2014).
18. Laronda, M.M., Burdette, J.E., Kim, J.J. & Woodruff, T.K. Recreating the female reproductive tract in vitro using iPSC technology in a linked microfluidics environment. *Stem Cell Res Ther* **4** (2013).
26. Zhu, J. et al. Human fallopian tube epithelium co-culture with murine ovarian follicles reveals crosstalk in the reproductive cycle. *Molecular human reproduction* (2016).

Reviewer #3

The current manuscript has investigated the potential of microfluidic culture systems to mimic the menstrual cycle hormone control of different tissues within the female reproductive tract.

They demonstrate that external stimulation of murine follicles or ovarian biopsy (with FSH and hCG) can not only induce follicular maturation but also the appropriate estrogen and progesterone profile required for endometriual growth and maturation. This model system has potential to investigate alterations in hormonal influences on different aspects of reproductive tract biology at different parts of the cycle within a system that takes into account the interconnected organs of the reproductive tract and their influence on each other.

Thank you for your comments on our study.

In general over the 28 days in culture tissue integrity was good, with the exception of endometrium which required reseeding of stromal cells and epithelial cells into cell free endometrial scafolds. However, the endometrial stroma contains a large number of leucocytes, numbers of which change dramatically thoughout the menstrual cycle, particularly in the mid-late secretory phase of the cycle. The exact role of these cells in endometrial biology are not known but they are proposed to play roles in implantation, menstruation, endometrial maturation etc. I accept that endometrial tissue does not do well in culture, even under near constant flow conditions. However, the absence of leucocytes in this experimental system needs to at least be commented on in the discussion as a potential limitation.

We appreciate reviewer's insight into the importance of other cell types of the endometrium. Indeed, leukocytes and in particular natural killer cells are recruited and play an active role in the remodeling of the endometrium and have been implicated to influence key physiological functions, including implantation, menstruation, remodeling as well as promote a pathological phenotype. Mice have been used to study the role of NK cells and human tissues have shown the changing incidence of these cells in various contexts. To date, there are limited in vitro systems to investigate cause and effect type of studies. The paracrine actions of leukocytes with endometrial cells have been studied in mixed monolayer cultures or transwell type systems (following references A-C). The decellularized endometrial scaffold provides a native tissue-like environment and structure for cells to interact and in future years, circulating cells like leukocytes could be envisaged to move between wells in the same way that the fluid dynamically flows. A brief discussion has been included in the revised manuscript.

A. Gong X[1], Liu Y[1], Chen Z[2], Xu C[1], Lu Q[1], Jin Z[1]. Insights into the paracrine effects of uterine natural killer cells. Mol Med Rep. 2014 Dec; 10(6): 2851-60. doi: 10.3892/mmr.2014.2626. Epub 2014 Oct 13.

B. Ho LS Tsang LL Chung YW Chan HC Establishment of a mouse primary co-culture of endometrial epithelial cells and peripheral blood leukocytes: effect on epithelial barrier function and leukocytes survival. Cell Biol Int 2006 30 977–82

C. Lebovic DI, Chao VA, Taylor RN. Peritoneal macrophages induce RANTES (regulated on activation, normal T cell expressed and secreted) chemokine gene transcription in endometrial stromal cells.J Clin Endocrinol Metab. 2004 Mar; 89(3): 1397-401.

In addition, it was not clear if glandular structures were reproduced in the reconstituted endometrium and whether they became functionally active.

Glandular structures were present in the decellularized scaffold. However, endometrial epithelial cells did not take residence in the glandular scaffold. Culturing epithelial cells has been a major limitation in our field and improvements in maintaining epithelial cells *in vitro* is needed. The use of stem cells may be a viable alternative. Thus, the cells that grew within the scaffolds were mostly endometrial stromal cells.

Reviewer #1 (Remarks to the Author)

The authors have properly addressed the major concerns raised in the first round of review. Publication of the revised manuscript is recommended without further requests for revision.

Reviewer #1 (Remarks to the Author):

The authors have properly addressed the major concerns raised in the first round of review. Publication of the revised manuscript is recommended without further requests for revision.

A: We thank reviewer #1 for the positive comment on our revised manuscript.
